# BMP7 Expression Correlates with Secondary Drug Resistance in Mantle Cell Lymphoma

**DOI:** 10.1371/journal.pone.0073993

**Published:** 2013-09-12

**Authors:** Valérie Camara-Clayette, Serge Koscielny, Sébastien Roux, Thierry Lamy, Jacques Bosq, Marc Bernard, Thierry Fest, Vladimir Lazar, Gilbert Lenoir, Vincent Ribrag

**Affiliations:** 1 Institut de Recherche Intégrée en cancérologie à Villejuif (IRCIV), Institut de cancérologie Gustave Roussy, Villejuif, France; 2 Institut National de la Santé et de la Recherche Médicale (INSERM) U1009, Université Paris Sud, Institut de cancérologie Gustave Roussy, Villejuif, France; 3 Biostatistics and Epidemiology Service, Department of Translational Research, Institut de cancérologie Gustave Roussy, Villejuif, France; 4 Service d’Hématologie, Centre Hospitalier Universitaire de Rennes, Rennes, F-35033, France; 5 Institut National de la Santé et de la Recherche Médicale (INSERM) UMR U917, Université Rennes 1, Rennes, France; 6 Département de Bio-Pathologie, Institut de cancérologie Gustave Roussy, Villejuif, France; 7 Département de Médecine, Institut de cancérologie Gustave Roussy, Villejuif, France; Faculty of Medicine, University of Porto, Portugal

## Abstract

**Purpose:**

We designed a gene profiling experiment to identify genes involved in secondary drug resistance in mantle cell lymphomas (MCL).

**Experimental Design:**

We obtained paired tissue samples collected from the same patients before treatment and after relapse or progression. Variations in gene expression between the 2 samples were estimated for 5 patients. For each gene, the mean variation was estimated for patients with a refractory primary tumor and for responders who developed secondary drug resistance. Nine genes of interest were selected on the basis of the magnitude and statistical significance of the variation of expression in responders and non-responders.

**Results:**

BMP7 was the only one with significantly increased expression at relapse in patients who developed secondary resistance. Validation of BMP7 as a key gene involved in secondary resistance was performed using cultures of cell line. Incubation of BMP7 with MCL cell lines increased their resistance to bortezomib and cytarabine, while inhibition of BMP7 expression by siRNA correlated with increased cell death linked to drug application.

**Conclusion:**

Variations in gene expression after treatment point out BMP7 as a key gene involved in secondary resistance in mantle cell lymphoma.

## Introduction

Mantle cell lymphoma is a rare lymphoma entity which is usually incurable with available therapies. Despite a high rate of response to 1^st^-line immuno-chemotherapy, the probability of cure is low and the median survival about 5 years [1]. In patients who achieve a primary response to therapy, secondary drug resistance almost invariably develops [2,3]. Identification of mechanisms involved in secondary drug resistance is a major challenge in MCL. The biological effects of therapy on tumor cells is an area of intense research in oncology. Microarray analyses offer the opportunity to identify genes that may be deregulated during tumor progression or after anticancer therapy. The current strategy of gene profiling is based on the use of tumor samples obtained exclusively before treatment. The objective of this strategy is to find genes or combinations of genes likely to predict the effect of treatment. Unfortunately, this strategy leads to unstable results and to poor quality predictions [4]. To overcome these problems, one solution is to increase the size of the studies; the price for credibility being the inclusion of tens or hundreds of patients. This solution is not well suited to rare pathologies. Moreover and paradoxically, the idea of using large series of patients may be justified only when the objective is to demonstrate that small difference between two groups are statistically significant, but may not be relevant for the development of an individual patient-oriented therapy. At the individual patient level small differences in gene expression have slim chances of being biologically relevant and clinically useful. This is why we focus on changes in gene expression (instead of pretreatment gene expression levels) that are large enough to be potentially biologically relevant and could be used as biomarkers at individual patient level (instead of being statistically significant in large groups of patients). We looked at changes in gene expression using paired tissue samples collected from the same patient, one sample taken before and the other after relapse or progression. We assumed that the variation in gene expression between the two samples from the same patient reflected modifications that occurred during tumor progression and could reveal new biological mechanisms of drug resistance. The findings observed through this strategy were further investigated using cell lines.

## Materials and Methods.

### Patients

Nineteen patients participated in the study. This study was approved by Institut gustave Roussy IRB, and data were analyzed anonymously. Paired frozen tissue samples were obtained from 12 patients and formalin-fixed and paraffin embedded (FFPE) tissue samples from seven other patients (Table 1).

**Table 1:** Patient characteristics; Tissue samples were collected from Patients 1 to 12 for microarray analyzes; the quality of one or 2 of the samples was insufficient for patients 6 to 12.

#	Gender	age	LDH	Stage	Treatment	response	Tissue origin:	Pre-treatment sample	Post-treatment sample
1	M	74	N	IV	CLB	**Yes**	**Lymph-node**	OK	OK
2	F	56	>N	IV	DHAP-R	**yes**	**blood**	OK	OK
3	M	73	N	IV	CHOP	**No**	**Lymph-node**	OK	OK
4	M	73	>N	IV	DHAP-R	**yes**	**blood**	OK	OK
5	M	63	N	IV	CLB	**No**	**blood**	OK	OK
6	M						lymph node	Quality pb	OK
7	M						lymph node	Quality pb	Quality pb
8	M						lymph node	Quality pb	Quality pb
9	M						Lymph node	Quality pb	Quality pb
10	M						blood	Quality pb	Quality pb
11	F						Blood	Quality pb	OK
12	F						blood	OK	Quality pb
13	M	50	N	IV	CHOP	No	**Lymph-node**	OK	OK
14	F	60	N	III	CHOP	yes	**Lymph-node**	OK	OK
15	M	58	>N	IV	CHOP-DHAP	yes	**Lymph-node**	OK	OK
16	M	63	N	IV	CHOP	No	**Lymph-node**	OK	OK
17	M	43	N	II	CHOP	No	**Lymph-node**	OK	OK
18	M	48	N	IV	CHOP	yes	**Lymph-node**	OK	OK
19	M	54	ND	IV	CHOP	No	**Lymph-node**	OK	OK

Formalin-fixed and paraffin embedded (FFPE) samples were collected from patients 13 to 19 for immunohistochemical studies.

LDH: Lactate deshydrogenase; N: normal is good prognostic factor, >N is poor prognosis factor

Quality pb: inadequate quality

ND: not done

### Tissue samples

All patients had 2 samples collected from the same tissue (blood or lymph nodes). One sample was collected before treatment and the second was collected at the time of relapse for patients who had initially responded to the treatment and subsequently developed secondary resistant disease, or at the end of initial therapy when the lymphoma was considered refractory to treatment (at least 4 weeks after the last chemotherapy).

### Microarray analysis

Microarrays were processed when both the pretreatment and the post-treatment tissue sample had an adequate quality of RNA. We used Agilent long (60 bp) oligonucleotide microarrays and the dual color analysis method in which probes from two specimens are differentially labeled by incorporating Cyanine 5 and Cyanine 3, respectively. For each patient, the paired sample (before treatment and at relapse/progression) was co-hybridized on Agilent dual color DNA chips.

The results of the microarray analysis have been deposited on array-express (http://www.ebi.ac.uk/arrayexpress/, Experiment name: IGR_CamaraClayette_MCL; ArrayExpress accession: E-MTAB-1090); the details for journal/reviewer access are Username: Reviewer_E-MTAB-1090; and Password: ULK4aVie.

### Cell culture

Jeko human lymphoma cell lines were cultured in RPMI 1640 medium and UPN1 human lymphoma cell lines were grown in alpha-MEM (Invitrogen, France).

### Bisulfite DNA treatment and sequencing

Genomic DNA was isolated from MCL cell lines using QIAGEN’s standard procedures. The converted *BMP7* promoter was identified by PCR with converted primers: forward (5′-GGATTTTTAGGTTTGTTGGTTG-3′) and reverse (5′ CAACTCACAATAAACACACATACAT-3′).

### Immunohistochemistry

Immunohistochemical studies of *BMP7* were performed on surgical specimens from patients with Mantle Cell Lymphoma using the avidin–biotin–peroxidase method (Quick kit, Vector Laboratories, USA) on formalin-fixed, paraffin-embedded tissue sections (4 µm). The mouse monoclonal antibody directed against human *BMP7* (R&D Systems, Inc., Minneapolis, MN ; Ref : MAB3542 ; clone 164313) was used at 25 µg/mL (1:40).

### SiRNA transfections and chemotherapy

The following double-stranded RNA 21 base pair oligonucleotides (Qiagen S.A, Courtaboeuf, France) were used: BMP7-3: 5’ CAA UGA ACA AGA UCC UAC A dTdT 3’ and 5’ UGU AGG AUC UUG UUC AUU G dGdA 5’ BMP7-4: 5’ CGG AAG UUC CUG UAA UAA A dTdT3’ and 5’ UUU AUU ACA GGA ACU UCC G dGdG 5’. Transfections were performed using 4 µl of lipofectamine 2000 (Invitrogen, France) incubated 10 minutes in a total volume of 100 µl of serum-free Opti-Mem. Chemotherapy was added to the medium 24 or 48 hours post-transfection over 24 hours. Cytarabine and Bortezomib were dissolved according to the manufacturer’s instructions before adding 20 µg/ml and 5 ng/ml respectively to cell culture media.

### Statistical analysis of microarray data.

Each gene was analyzed independently of the others. The main analysis was based on expression log-ratios, which were defined as the natural logarithm of the ratio between the expression measured in the sample obtained after treatment and the expression in the sample collected before treatment from the same patient.

## Results

Paired frozen tissue samples were obtained from 12 patients. The RNA quality was sufficient for microarray analyses for only 5 out of the 12 patients initially screened for the microarray analyses (Table 1). Of the 5 patients with both RNA samples eligible for the analyses (one collected before treatment and one after treatment), 3 were initially sensitive to chemotherapy and relapsed after developing secondary resistance (secondary resistant tumor) and 2 had a refractory primary disease and did not respond to their first treatment (non-responders).

**Figure 1:**
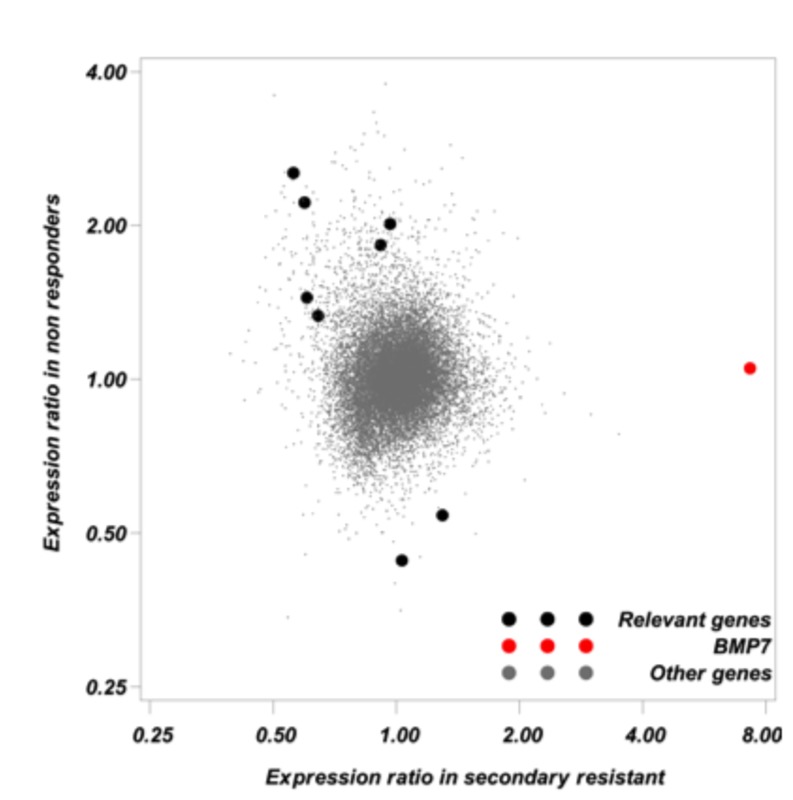
Gene expression ratios were calculated for each gene in primary refractory (non-responders) and in responders (secondary resistant). Gene expression ratio is equal to the geometric mean gene expression after treatment divided by the geometric mean gene expression before treatment. Gene expression ratios greater than one correspond to increased expression after treatment and conversely. Each point on the figure corresponds to a gene expression ratio in responders according to the expression ratio in non-responders. Nine genes classified as relevant (see methods) are symbolized by black dots. BMP7 which was selected for further investigations is symbolized by a red dot. Small grey symbols were used for “Other genes”.

Gene expression fold change was defined as the ratio between the expression after therapy and at diagnosis. For each patient, the paired sample (before treatment and at relapse/progression) was co-hybridized on Agilent dual color DNA chips. The main analysis focused on the ratio between gene expression fold change in initially sensitive (secondary resistant tumor) and primary refractory tumors (non-responders) or fold change ratio (FCR). Nine genes with an absolute FCR value greater than 2 and a P value <0.01 were considered relevant (Table 2). BMP7 expression, which was very low before treatment in all patient’s samples whatever the clinical response, was multiplied by 7.31 after relapse in secondary resistant tumors, but did not vary significantly in non-responders (Figure 1). This represents the largest absolute FCR value of the 9 relevant genes (FCR=6.96; P=0.002). Most of the other relevant genes had significantly decreased expression at relapse in secondary resistant tumors and/or increased expression in non-responders. Because BMP7 had an increased expression at relapse in secondary resistant tumors, we investigated the putative role of BMP7 in secondary resistance.

**Table 2:** Characteristics of the genes with a relevant fold change ratio (FCR).

Short name / Description		Expression ratio in non-responders (A)		Expression ratio in responders (B)		Fold Change Ratio FCR(*)	
		Value	P value	Value	P value	Value	P value
BMP7	Bone morphogenetic protein 7, regulates cell proliferation, cell differentiation, apoptosis, folliculogenesis, ovulation, and bone formation, may play roles in skeletal and nervous system development, preserves kidney function after acute renal failure	1.05	0.76	7.31	0.0005	6.96	0.002
EGR3	Early growth response 3, member of the Egr family of immediate early transcription factors, induced by light in the suprachiasmatic nucleus (SCN); deletion of murine Egr3 results in higher perinatal mortality and multiple muscle development disorders	2.54	0.002	0.56	0.005	-4.53	0.001
BCL2A1	BCL2-related protein, a member of the Bcl-2 family of apoptosis regulators; inhibits apoptosis, promotes tumorigenesis, and may play a protective role during inflammation	2.21	0.005	0.60	0.009	-3.72	0.002
I_1000049	Protein of unknown function	0.54	0.009	1.30	0.05	2.39	0.007
TBX21	Protein with strong similarity to T-box 21 (mouse Tbx21), which is a transcription factor that likely regulates differentiation of helper T-cells into Th1 cells, contains a T-box domain, which bind DNA	1.45	0.03	0.60	0.008	-2.39	0.007
TRAF5	TNF receptor-associated factor 5, member of a family of proteins that interact with the cytoplasmic domain of oligomerized TNF receptors, binds CD40 (TNFRSF5) and mediates signaling through activation of NF-kappaB and JNK pathways	0.44	0.002	1.03	0.66	2.33	0.004
CCL28	*Homo sapiens* chemokine (C-C motif) ligand 28 (CCL28), transcript variant 2, mRNA	2.02	0.0005	0.97	0.39	-2.09	0.001
RAB11-FIP4	Protein with moderate similarity to human Rab11-FIP3, which functions in vesicle-mediated transport	1.33	0.05	0.64	0.009	-2.07	0.008
MGC4189	Protein of unknown function	1.83	0.001	0.92	0.02	-2.00	0.002

FCR is estimated as B/A. When the ratio is less than 1, FCR value is given as – A/B. The table is sorted according to the absolute value of FCR.

### Expression of BMP7 at relapse in MCL patients initially sensitive to chemotherapy

**Figure 2:**
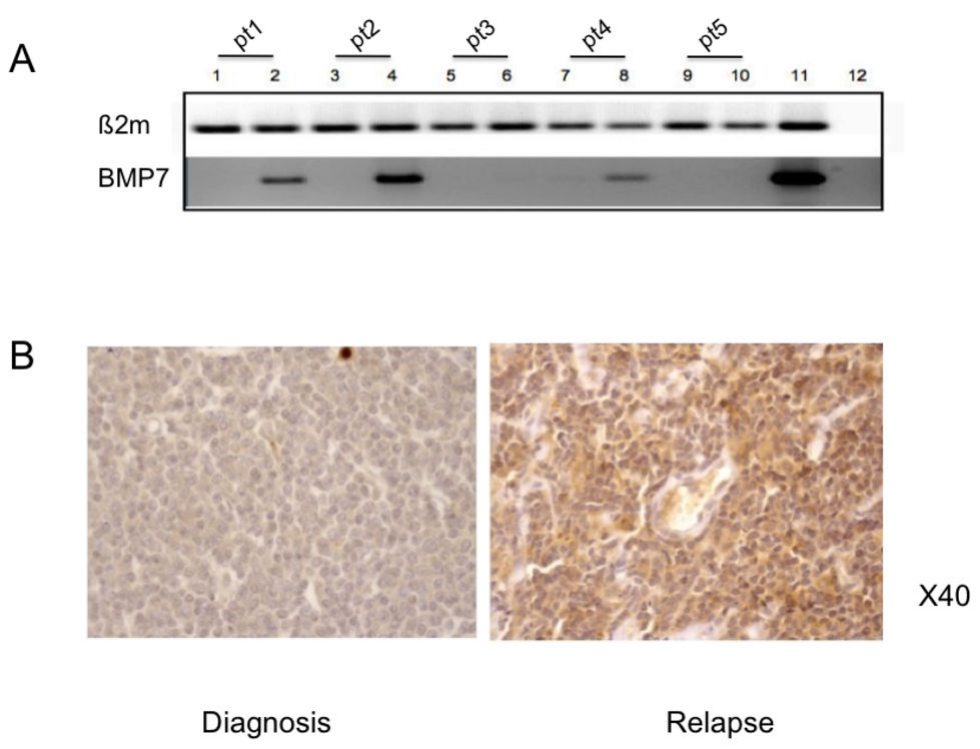
Expression of BMP7 in MCL lymphoma cells. Figure 2A: from patients tested by DNA chips, RT-PCR was performed in two independent experiments before therapy (lane 1, 3, 5, 7 and 9) and after therapy (lane 2, 4, 6, 8 and 10). Patient 1 (lanes 1 and 2), patient 2 (lanes 3 and 4), patient 3 (lanes 5 and 6), patient 4 (lane7 and 8) patient 5 (lanes 9 and 10). Patients 1, 2 and 4 were initially sensitive to chemotherapy (secondary resistant tumors). Human placenta was used as a positive control (lane 11) and mix without cDNA as negative control (lane 12). BMP7 was expressed in the 3 patients at relapse, but was not expressed in the 2 patients with refractory disease at diagnosis and after having failed on therapy. ß2microglobuline probe was used as internal reference. Figure 2B: Immunohistochemical analysis of one of the 2 patient’s tumor at diagnosis and at relapse, that expressed BMP7 at relapse (secondary resistant lymphoma).

The presence of BMP7 mRNA in the samples of the 5 MCL patients tested on Agilent DNA chips was verified with RT-PCR analyses. BMP7 was detectable only in the 3 samples obtained at the time of relapse in secondary resistant tumors; it was not detectable in the samples obtained before treatment whatever the response to initial therapy, nor in the samples obtained at relapse in refractory primary tumors (Figure 2A).

We also assessed whether the BMP7 protein could be detected by immunohistochemistry on paraffin embedded tissues in MCL cases (none of these cases had frozen samples for PCR analysis). Seven paired samples collected at diagnosis and after first-line therapy (patients 13 to 19, Table 1) that were not included in the Agilent DNA chips assays were investigated: 3 secondary resistant tumors (initially sensitive) and 4 refractory primaries diseases (non responders). None of these 7 patient samples expressed BMP7 at diagnosis, 2 out of 3 secondary resistant samples expressed detectable BMP7 at relapse (see Figure 2B that represent one of the two BMP7-positive patient at relapse), and none of the 4 refractory primary tumors expressed BMP7 during the refractory phase of the disease

Overall, of the 12 MCL samples analyzed at diagnosis, none had BMP7 expression detectable by gene array or IHC analysis. Out of the 6 initially sensitive patients, 5/6 tumor samples collected after treatment had an increase of BMP7 expression. None of the 6 samples collected from non-responders patients after treatment expressed BMP7.

### BMP7 expression in MCL cell lines

**Figure 3:**
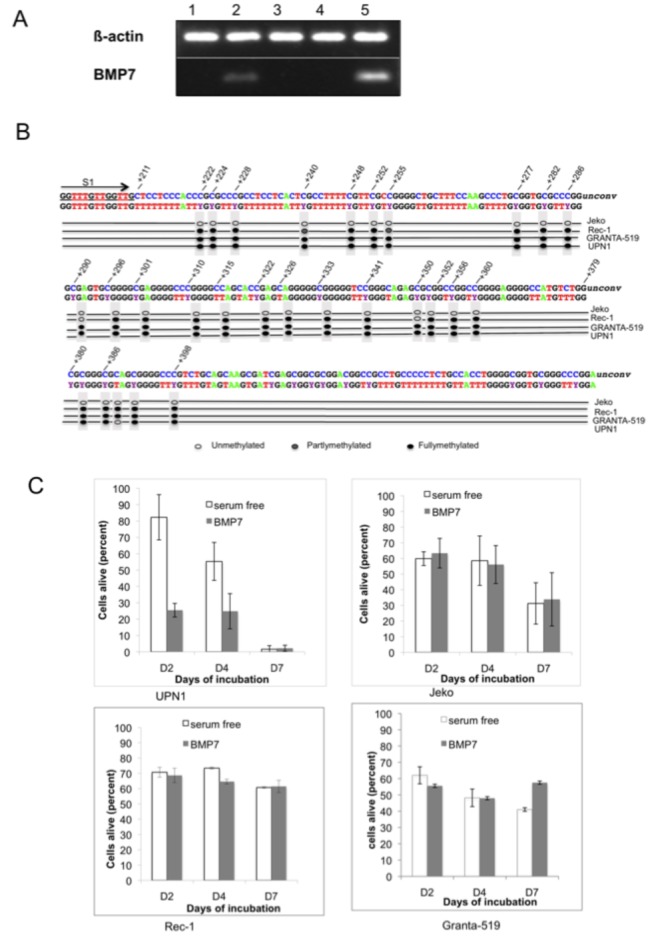
A: Expression of mRNAs encoding BMP7 tested by RT-PCR performed on 1: UPN1, 2: Jeko, 3: Rec-1, 4: GRANTA-519 and 5: placenta (positive control). B: methylation profile of BMP-7 CpG islands in the cell line C: UPN1, Jeko, Rec-1 and GRANTA-519 cells were cultured in serum-free media in the presence or absence of 200 ng/ml of BMP-7 for 7 days. BMP-7 had no effect on Jeko, GRANTA-519 and Rec-1 cell survival and on annexin V-positive cells compared to serum-free media alone.

To test the hypothesis that BMP7 was supposedly a biological factor capable of protecting MCL cells against chemotherapy, we investigated with RT-PCR analysis whether BMP7 mRNA was detectable in four MCL cell lines (UPN1, Jeko, Rec-1 and GRANTA-519). Jeko expressed BMP7 mRNA, but the other cell lines tested did not (UPN1, Rec-1 and GRANTA-519) (Figure 3A). We used an Elisa assay to investigate whether Jeko cells were able to secrete BMP7. BMP7 was detectable in the supernatant of Jeko cells at a concentration exceeding 10 pg/ml on day 7 of culture in serum-free Opti-Mem medium, whereas no BMP7 was detected in medium alone (optical density lower than the negative control of the scale) as well as in the supernatant from UPN1 on days 2 and 7, or Jeko on day 2.

Since BMP7 mRNA expression was different between the MCL cell lines, we investigated the methylation profile of the BMP7 promoter CpG islands in the four MCL cell lines. The BMP7 promoter was found to be hypermethylated in UPN1, Rec-1 and GRANTA-519 cell lines but was demethylated in the Jeko cell line (Figure 3B).

### Protective effect of BMP7 inexcess against Bortezomib on Jeko cells

The BMP7 effect on cell survival was measured after 2 days incubation in the presence of BMP7 in excess (200 ng/ml). BMP7 in excess had an apoptotic effect on UPN1 cell line: the proportion of annexin V-positive cells was 14% in serum-free media and 74% in the presence of BMP7 (four independent experiments in triplicate P<10^-4^). No effect of BMP7 in excess was found on Jeko, Rec-1 and GRANTA-519 survival, even when exposure was extended to 7 days (Figure 3C). We assessed whether BMP7 had a protective effect against Bortezomib cytotoxicity on BMP7-expressing Jeko cell lines. Jeko cells were cultured with BMP7 (200 ng/ml) or in free media conditions. Jeko cells treated with Bortezomib plus BMP7 had a lower apoptotic cell rate when compared to the same cells cultured in the same conditions but without BMP7 (four independent experiments in triplicate; P=0.002, Mann Withney test; data not shown)

### Effect of BMP7 mRNA inhibition on Bortezomib and Cytarabine cytotoxicity

**Figure 4:**
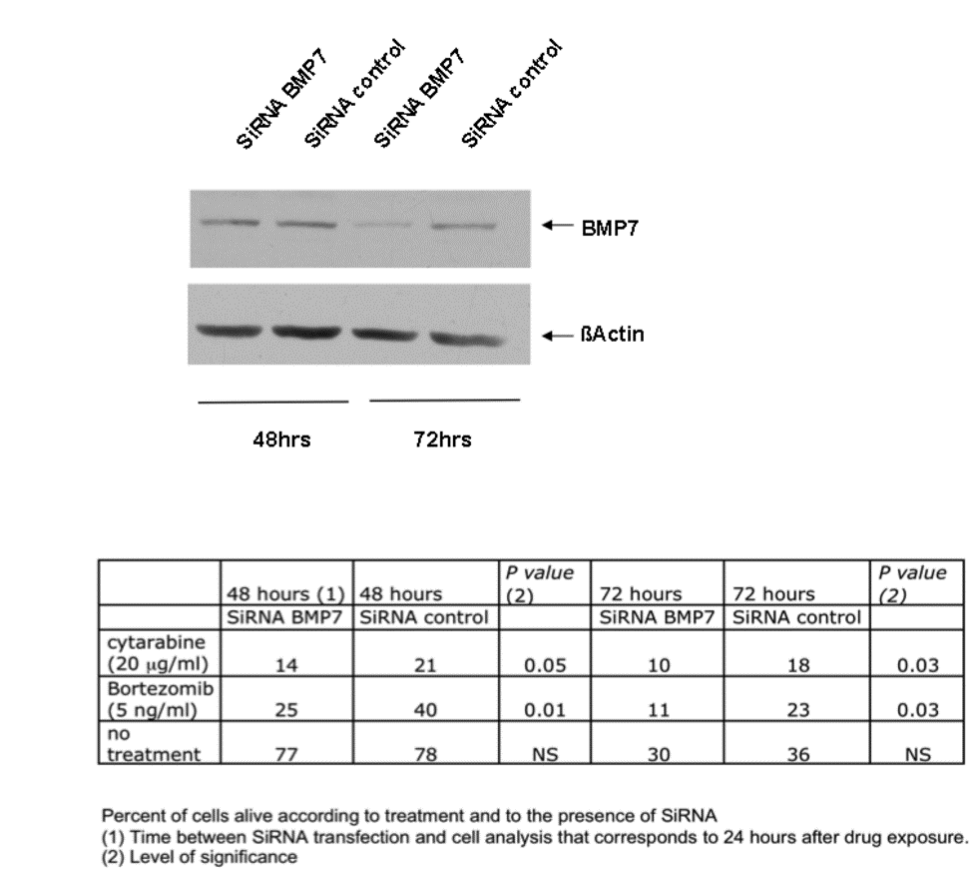
SiRNA BMP7 had a negative effect on cell survival at all time points assessed with both drugs showing that BMP7 plays a role in drug resistance. Jeko cells were exposed with Bortezomib (5 ng/ml) or Cytarabine (20 µg/ml) 24h or 48h after transfection with a BMP7 siRNA or a nonsilencing siRNA used as a control (see material and methods). BMP7 protein expression was assessed by immunoblotting analyzes. Annexin V- and PI-negative cells (non apoptotic) were quantified 48h and 72h after transfection. BMP7 siRNA markedly increased the fraction of annexin V- and PI-negative cells showing that BMP7 suppression plays a role in Jeko cell chemosensitivity to Bortezomib and Cytarabine.

Jeko cells were transfected using a transient transfection assay with two BMP7 siRNAs (BMP7-4 and BMP7-3) or a nonsilencing siRNA as a control. At 48h and 72h post transfection with BMP7-4 siRNA, the mRNA levels of BMP-7 were reduced by 55 and 57% respectively and the expression of the protein at 72 hours (Figure 4). No direct effect on cell survival was observed neither with the BMP-7 siRNA nor with the nonsilencing siRNA at 48h and 72h after cell transfection. We next treated Jeko cells 24h or 48h after transfection with either Bortezomib (5 ng/ml) or Cytarabine (20 µg/ml) for 24 h. Drugs were given at their IC50 concentration on Jeko cells (data not shown). Apoptotic cells were quantified 48h and 72h after transfection. Cytarabine and Bortezomib induced apoptosis in approximately 66% and 60% of cells (measured as annexin V- and PI-positive cells) at 48h respectively. When cells were transfected with BMP7 siRNA, the proportion of apoptotic dead cells (annexin V- and 7-AAD-positive cells) was markedly increased (from 8.3% to 31.5%; P = 0.002) when exposed to Bortezomib at 5 ng/ml. This increased apoptotic rate was also associated with a decreased number of viable cells (Figure 4). The same experiments were performed with the BMP7-3 siRNA with similar results (data not shown).

## Discussion

Using tissue from the same patient’s tumor collected at different phases of the disease is an attractive option since the internal control for gene array analysis is achieved with the same tumor. The sample at diagnosis is collected from the same patient as the sample analyzed at relapse or at the refractory phase of the disease. Our approach is based on the analysis of gene expression changes that occurred between initial treatment and relapse, when secondary resistance developed. We arbitrarily chose to exclusively select and investigate genes with at least a two-fold change in their expression. We identified BMP7 based on data from a limited number of patients assessed at different time points during their treatment, using very stringent gene selection criteria. We then used cell lines to validate the results. BMP7 was the only gene with a relevant increase in expression at relapse, but not in refractory cases. Our studies on cell lines are consistent with better resistance of BMP7-positive cell lines to cytotoxic agents or targeted therapy. The limited number of patients is a drawback essentially due to working on a rare and molecularly well-defined pathology, but also on collection of two independent biopsies with a good quality mRNA sampling from the same patient. Even in the case of reaching high quality samples for gene array analysis in 80% of biopsies, requiring two biopsies from the same patient decrease this probability to about 60% [5]. However, we consider that dealing with small sample sizes is a mandatory step on the road to personalized medicine, where every patient is expected to gain access to personalized treatment. This is contrary to what is done in most (past and present) profiling studies performed on heterogeneous pathologies and in which the possibility of a unique molecular mechanism is improbable. The strategy we used in this work differs with classic gene expression profiling in at least 3 respects: patients were used as their own controls; gene selection was performed at the patient’s level and considered relevant when it was found in other patient’s samples collected in the same clinical situation, and findings were ultimately validated biologically.

Of the different genes selected with this strategy, BMP7 was the only gene that was expressed only at relapse and in patients who initially responded to therapy. Our findings suggested that BMP-7 could be a gene involved in secondary drug resistance in these mantle cell lymphomas. To our knowledge, no other studies focused on a potentially protective effect of BMP7 in cancer cells in stressed conditions have been reported. We found that BMP7 down regulation in Jeko cells was able to increase anticancer drug sensitivity. Furthermore, an excess of BMP7 had a protective effect when the same cells were treated with cytarabine or bortezomib.

This is the 1^st^ time that BMP7 is identified as a candidate gene that might play a role in secondary drug resistance. Little is known about the role of BMP7 in oncology, especially in drug resistance. BMP proteins (BMPs) are secreted signaling molecules that belong to the transforming growth factor superfamily [6]. BMP7 signaling is elicited through specific serine/threonine kinase receptors. Initially, BMP7 interacts with BMP receptor R2 which subsequently binds to BMP receptor R1. This BMP R1-R2 dimerization induces activation of the canonical BMP pathway through smad 1, 5 and 8 phosphorylation which forms heteromeric complexes with the common mediator smad 4. Activated smad complexes then translocate into the nucleus and bind to DNA consensus smad binding sites and directly regulate the expression of different target genes [7–9]. Like many other RTK, the activated BMP receptor complex is now known to activate alternative pathways independently of the Smad signaling pathway. These alternative effects include activation of the phosphoinositol-3 kinase (PI3K) through PTEN inhibition and activation of mitogen activated protein kinases (MAPK) through extracellular signal regulated kinase (ERK), c-jun N-terminal kinase (JNK), and p38 [10–12]. The role of BMP7 in oncogenic processes was recently investigated in various tumor models [12–18]. To our knowledge, no other study has reported on the role of BMP7 in primary or secondary drug resistance, and recent studies shown that BMP7 was the most frequently express BMP protein in lymphoma cells and in normal germinal center B-cells [19]. Nevertheless, in multiple myeloma BMP6 was found to protect cells against bortezomib-induced apoptosis [20]. Although BMP7 induce apoptosis in normal B-cells, B-cell lymphoma cells can escape BMP-induced inhibition of cell growth suggesting an autocrine growth regulation and that malignant cells can escape autocrine growth inhibitory effect of BMP7 [19,21]. However, in these studies, the role of BMP7 in secondary drug resistance was not explored. BMP7 expression was found to be associated with tumor progression and the occurrence of metastasis in malignant melanomas and in colonic carcinoma [15,22]. The effect of BMP7 on cell survival has been studied essentially in prostatic tumors. In the C4-2B prostatic cancer cells, BMP7 is able to promote cell survival by inhibiting stress-induced apoptosis in culture-free medium conditions via both the smad/survivin and c-jun NH2-terminal kinase pathway putatively through survivin overexpression [12].

We found that there was a relation between promoter hypermethylation and BMP7 mRNA expression in MCL cell lines. The same epigenetic regulation of BMP7 was also observed in prostatic and gastric cancers where BMP7 expression is associated with a more aggressive phenotype [14,23,24].

In conclusion, secondary drug resistance could be the result of different processes and remain an important question in oncology. One hypothesis in MCL could be that, before therapy, BMP7-positive cells had a proliferation disadvantage compared to BMP7-negative cells that compose the majority of tumor cells, and would be undetectable but present in the MCL. In initially chemo sensitive cases, active chemotherapeutic agents kill the BMP-7 negative cells, leading to the persistence of selected BMP7-positive cells that are more prone to resist to chemotherapy. At relapse, these BMP7-positive cells may represent the majority of tumor cells and became detectable. In primary chemoresistant lymphoma, these cells remained undetectable. This could probably explain why BMP7 was not detectable in the majority of lymphoma cells from patients with primary resistant MCL (before treatment and at the time of progression). This strategy is not adapted to identify relevant biological targets of refractoriness in primary refractory cases where no major modification is observed in the tumor over time (when we compare the tumor at diagnosis and in the refractory phase after one line of chemotherapy as it was the case in this study). We are able to identify modification that appears in the tumor where chemotherapy is able to induce major reduction of tumor burden. Our results suggest that with a simple and sequential gene array evaluation of tumors, new genes of interest can be identified in a few, but well characterized and homogeneous groups of tumors.

## Supporting Information

File S1 **Supporting materials and methods.**
(DOC)
