# Supplementary material for: BMP7 Expression Correlates with Secondary Drug Resistance in Mantle Cell Lymphoma
Source: PLoS One. 2013 Sep 12;8(9):e73993. doi: 10.1371/journal.pone.0073993 (PMC3771972; doi:10.1371/journal.pone.0073993)
Supplement: File S1 — Supporting materials and methods. (DOC) [file pone.0073993.s001.doc]

**Materials and Methods S**

Patients

Nineteen patients participated in the study. All patients gave their informed consent. Paired frozen tissue samples were obtained from 12 patients and formalin-fixed and paraffin embedded (FFPE) tissue samples from seven other patients (Table 1).

Evaluation of treatment response

Response to therapy was defined as a decrease of 50% or more in the sum of the products of the maximum perpendicular diameters of all measured lesions that persisted for at least four weeks. Treatment failure was defined as no response.

Tissue samples

All patients had 2 samples collected from the same tissue (blood or lymph nodes). One sample was collected before treatment and the second was collected at the time of relapse for patients who had initially responded to the treatment and subsequently developed secondary resistant disease, or at the end of initial therapy when the lymphoma was considered refractory to treatment (at least 4 weeks after the last chemotherapy).

RNA preparation and quality control

Total RNAs were prepared with Sigma’s Tri-reagent protocol and cleaned up using the Rneasy minikit from Qiagen according to the manufacturer instructions. The quality of RNAs was assessed using lab-on-a-chip Bioanalyser technology including an electropherogram.

Microarray analysis

Microarrays were processed when both the pretreatment and the post-treatment tissue sample had an adequate quality of RNA. We used Agilent long (60bp) oligonucleotide microarrays and the dual color analysis method in which probes from two specimens are differentially labeled by incorporating Cyanine 5 and Cyanine 3, respectively. For each patient, the paired sample (before treatment and at relapse/progression) were co-hybridized on Agilent dual color DNA chips. A first round of experiments was carried on Agilent 22k microarrays (Agilent G4110A, AMADID 011521), in duplicate and dye swap replicates (4 microarrays per paired sample). A second round of dye swap experiments was performed in dye swap on Agilent human genome 22k microarrays (Agilent G4110B, AMADID 012097). Probes were obtained from 500 ng total RNA in two steps: double stranded cDNA using MMLV reverse transcriptase followed by T7 RNA polymerase linear amplification and labeling. One µg of purified Cy3-labeled cRNA from one sample was mixed with the same amount of the other Cy5-labeled sample from the same patient. Mixed cRNAs were hybridized to the microarrays for 17 hours at 60 °C, then washed with 0.6X and 0.01X SSC buffers containing triton, and dried with a nitrogen gun. Microarrays were scanned using the Agilent DNA microarray scanner.

Gene expression analysis

Total RNA from MCL cell lines were Reverse transcript poly (A) using Superscript II reverse transcriptase (Invitrogen, life technologie). PCR reactions were performed using Taq platinum (Invitrogen). β-actin or ß2microglobulin were used as an internal control. Commercialise Human normal cDNA from placenta was used as positif control (Invitrogen, life technologie). PCR products were detected by ethidium bromide staining on a 1.5% agarose gel and visualized using a The Molecular Imager® Gel Doc (Biorad, France).

Cell culture

Jeko human lymphoma cell lines were cultured in RPMI 1640 medium (Invitrogen, life technologie) supplemented with 20% FBS (Perbio Science, Brebieres, France), penicillin streptomycin 1%, L-glutamine 1% (Invitrogen, life technologie) at 37°C and 5% CO2. UPN1, GRANTA-519 and Rec-1 human lymphoma cell lines were grown in alpha-MEM (Invitrogen, life technologie) supplemented with 10% FBS, penicillin streptomycin 1%, L-glutamine 1% (Invitrogen, life technologie) at 37°C in the presence of 5% CO2.

Jeko, GRANTA-519 and Rec-1 were a generous gift from European research MCL network partner and UPN1 was generated at Institut Gustave Roussy.

Annexin V staining and Human BMP-7 immunoassay

10^6^ Jeko or UPN1 cells were plated in 1 ml of serum free medium (Opti-MEM ) with or without 200 ng/ml of recombinant human BMP-7 (R&D system) and incubated during 2, 4 and 7 days. Apoptotic and living cells in all samples were quantified by flow cytometry with FITC-labeled annexin V and propidium iodide (Beckman Coulter).

After incubation, cells were collected and centrifugated at 1200 rpm during 10 minutes. Supernatants were retained and cells were resuspended in 100µl of 1X binding buffer with annexin-V-FITC (Beckman Coulter) (1µl). The cells were incubated at 4°C for 15 min, and 150µl of 1X binding buffer was then added. Propidium iodide (250µg/ml stock solution PI) was added to the cells and annexin-V staining was analyzed using a Becton Dickinson flow cytometer and BD software acquisition and analysis programs.

The Quantikine human BMP-7 immunoassay (R&D system) was used to measure human BMP-7 in the supernatant from Jeko and UPN1 samples according the manufacturer’s instructions.

Bisulfite DNA treatment and sequencing.

Genomic DNA was isolated from MCL cell lines using QIAGEN’s standard procedures. Two hundred nanograms of total genomic DNA were modified by bisulfite treatment according to the manufacturer’s instructions (MethylDetector, Active Motif). The converted *BMP7* promoter was identified by PCR with converted primers: forward (5′-GGATTTTTAGGTTTGTTGGTTG-3′) and reverse (5′CAACTCACAATAAACACACATACAT-3′). The direct sequencing reaction was performed using standard conditions according to the manufacturer’s instructions (Applied Biosystems). Immunohistochemistry
Immunohistochemical studies of *BMP7* were performed on surgical specimens from patients with Mantle Cell Lymphoma using the avidin–biotin–peroxidase method (Quick kit, Vector Laboratories, USA) on formalin-fixed, paraffin-embedded tissue sections (4 µm). After deparaffinization, epitopes were restored in a 10 mmol/L citrate buffer (ph 7,3) by submitting them for 30 mn in water bash and blocking. The antigen–antibody reaction was carried out for 1h. The detection kit used was the Quick kit. The mouse monoclonal antibody directed against human *BMP7* (R&D Systems, Inc., Minneapolis, MN ; Ref : MAB3542 ; clone 164313) was used at 25 μg/mL (1:40).

Antibodies and Western immunoblots

Total extracts were prepared from MCL UPN1 and Jeko cell lines. Cells were washed with phosphate-buffered saline (1XPBS) and lysed in buffer (10 mM Tris HCl, pH 7.4; 150 mM NaCl; 1% Nonidet P40 (NP40); 1 mM EDTA (ethylenediaminetetraacetic acid)) supplemented with protease (Roche Diagnostics, Meylan, France) and phosphatase inhibitors (1mM NA_3_VO_4_). Lysates were cleared of debris by centrifugation at 12 000 rpm for 15 minutes at 4°C. Protein (30 µg) from total extracts was resolved by SDS-10%PAGE and transferred to a nitrocellulose membrane (Hybond, GE healthcare). The blot was blocked in 0.1 % Tween-PBS containing 5% nonfat dry milk. Rabbit polyclonal anti-human BMP7 (AbC117-3744) was purchased from AbCys S.A, Paris 10ème, France).

SiRNA transfections and chemotherapy

The following double-stranded RNA 21 base pair oligonucleotides (Qiagen S.A, Courtaboeuf, France) were used:BMP7-3: 5’CAA UGA ACA AGA UCC UAC A dTdT 3’ and 5’ UGU AGG AUC UUG UUC AUU G dGdA 5’BMP7-4: 5’CGG AAG UUC CUG UAA UAA A dTdT3’ and 5’ UUU AUU ACA GGA ACU UCC G dGdG 5’. JEKO cells were washed and resuspended at a final concentration of 5.10^5^ in 400 µl of serum-free Opti-Mem in 12-well plates. Cells were transfected with 100nM of one or the other double-stranded RNA (siRNA) previously described, directed against BMP7 RNA or 100nM of double-stranded RNA all star negative control. Transfection (1 experiment in triplicate) was performed using 4µl of lipofectamine 2000 (Invitrogen, France) incubated 10 minutes in a total volume of 100µl of serum-free Opti-Mem. 0.6µl of MATra-si reagent (IBA technology, SPIBIO, Montigny Le Bretonneux, france) was added, incubated over 10 minutes and mixed with cells. The negative control was nonsilencing siRNA with no significant homology with any known mammalian gene (Qiagen S.A, France). Chemotherapy was added to the medium 24 or 48 hours post-transfection over 24 hours. Cytarabin and Bortezomib were dissolved according to the manufacturer’s instructions before adding 20 µg/ml and 5ng/ml respectively to cell culture media.

Statistical analysis of microarray data

Each gene was analyzed independently of the others. The main analysis was based on expression log-ratios, which were defined as the natural logarithm of the ratio between the expression measured in the sample obtained after treatment and the expression in the sample collected before treatment from the same patient. Expression log-ratios were analyzed with a generalized linear model (GLM procedure, SAS version 9.1; Cary NC). The dependent variables were the status of the patient (refractory primary: no response to initial chemotherapy or secondary resistant: patient who had responded to initial therapy but who had relapsed). The six log-ratio values that were available for each patient were considered as repeated measurements of the same biological samples or technical replicates. The differential impact of treatment on gene expression according to the patient’ status was summarized by the Fold Change Ratio (FCR). The FCR was estimated according to the difference between the least square mean log-ratio in responders minus the least square mean log-ratio in non responders. When the difference was positive, the FCR was expressed as the exponential (exp) of the difference. When the difference was negative, the FCR was expressed as – exp(-difference). The absolute value of the FCR corresponds to the intensity of the biological effect and the FCR sign indicates the up or down regulation of genes that appeared after treatment in patients who had developed secondary resistance compared to non-responders (resistant primary tumors).
